# Classic and Non-Classic Features in Pseudohypoparathyroidism: Case Study and Brief Literature Review

**DOI:** 10.7759/cureus.1878

**Published:** 2017-11-26

**Authors:** Aaron R Kuzel, Muhammad Uzair Lodhi, Mustafa Rahim

**Affiliations:** 1 Department of Emergency Medicine, Lincoln Memorial University-Debusk College of Osteopathic Medicine; 2 Medical Student, Department of Medicine, Raleigh General Hospital, Beckley, Wv; 3 Assistant Clinical Professor of Internal Medicine, West Virginia University School of Medicine

**Keywords:** concomitant hypothyroidism, autosomal dominant pseudohypoparathyroidism, concomitant carpal tunnel syndrome, g protein coupled receptor, sensorineural hearing loss, albright hereditary osteodystrophy, chromosome 20q13.3, increased hyperparathyroid hormone, hypocalcemia, hyperphosphatemia

## Abstract

Pseudohypoparathyroidism is a rare condition that is due to a defect in the stimulatory G-protein coupled receptor, resulting in end-organ resistance to parathyroid hormone. Hereditary forms of pseudohypoparathyroidism present with certain classic features such as obesity, short stature, brachydactyly, and intellectual disability. Constellation of these physical features is known as Albright's hereditary osteodystrophy. In this case, 41-year-old male presented with the classic features of pseudohypoparathyroidism and with 59 lbs weight gain over six months. It was determined that the cause of the patient’s weight gain was due to concomitant hypothyroidism, which is a common association. There are several non-classic features and associated pathologies associated with pseudohypoparathyroidism. These conditions should be regularly screened for and assessed when a patient presents with pseudohypoparathyroidism.

## Introduction

Pseudohypoparathyroidism is a rare condition that has an incidence of 0.79 per 100 patients [1]. It is divided into type Ia, Ib, Ic, and type II, with the most common form being Ia. Type Ia and Ib are caused by maternally-inherited changes in complex imprinted GNAS locus at 20q13.32, resulting in molecular dysfunction of the α subunit in the stimulatory G-protein coupled receptor [2]. Hereditary forms of pseudohypoparathyroidism are present with a group of clinical features, referred to as Albright's hereditary osteodystrophy. Albright's hereditary osteodystrophy presents with classic findings such as brachydactyly, varying degrees of intellectual disability, obesity, round facies, subcutaneous ossifications, and short stature [1]. The exact mechanisms on molecular level are not completely understood. Patients with pseudohypoparathyroidism often experience concomitant hypothyroidism, especially those with type Ia, which is typically diagnosed in the neonatal period or later in the life of a patient. The theory behind this relationship is that the thyroid-stimulating hormone (TSH) receptor also relies on the use of a stimulatory G-protein coupled receptor, which is coded by the GNAS protein [3].

## Case presentation

A 41-year-old male patient presented to the office with his mother for a weight gain of 59 lbs over the period of six months and increasing shortness of breath. He is an established patient in the practice and was not in acute distress. The patient did not mention any chest pain, chest pressure or heaviness in the chest. He denied any new medication and stated that he has been taking his anti-hypertensive medications as advised. His review of systems was additionally negative for any headaches, dizziness, blurred vision, decrease in urine output, neck stiffness, fever, rash, difficulty with speech, swallowing or gait, diarrhea, and cough. No goiter, lethargy or heat/cold intolerance was mentioned. The patient has a past medical history of hypertension, autosomal dominant pseudohypoparathyroidism, and hypothyroidism. In addition, his mother and his brother both have pseudohypoparathyroidism, but his mother denied hypothyroidism. Patient also reported a history of sensorineural hearing loss and carpal tunnel syndrome. Physical examination revealed no lymphadenopathy, thyromegaly, or any thyroid nodules. Cardiac examination exhibited an audible S1 and S2 with regular rate and rhythm. No murmur or gallop was noted. Pulmonary examination revealed lungs clear to auscultation bilaterally. The lower extremities exhibited 2+ to 3+ bipedal edema bilaterally, but peripheral pulses were present and symmetric.

Given the patient was living a sedentary lifestyle, the patient was ordered a venous doppler, an echocardiogram to assess left ventricular function, and brain natriuretic peptide (BNP) levels. Electrocardiogram was performed in the office and exhibited a normal PR interval of 176 ms and an 86 ms QRS complex. No ST segment changes were appreciated. Laboratory exams exhibited normal BNP levels, but TSH level was elevated to 11.390 mIU/L (normal reference range is 0.4 ‑ 4.0 mIU/L). Venous doppler and echocardiogram studies were unremarkable. Patient’s calcium levels were severely depressed and previous basic metabolic panel studies indicated consistent hypocalcemia between the ranges of 6.9 to 8.5 mg/dL. Patient's previous laboratory studies also showed normal vitamin D levels, with normal or slightly elevated parathyroid hormone levels.  

## Discussion

**Figure 1:**
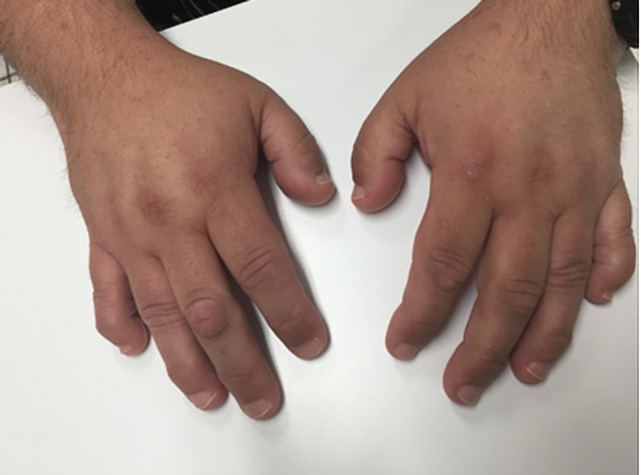
Brachydactyly of the hands Shortened first and fifth digits in both hands were observed on physical examination. Brachydactyly is considered a classic finding of pseudohypoparathyroidism.

**Figure 2:**
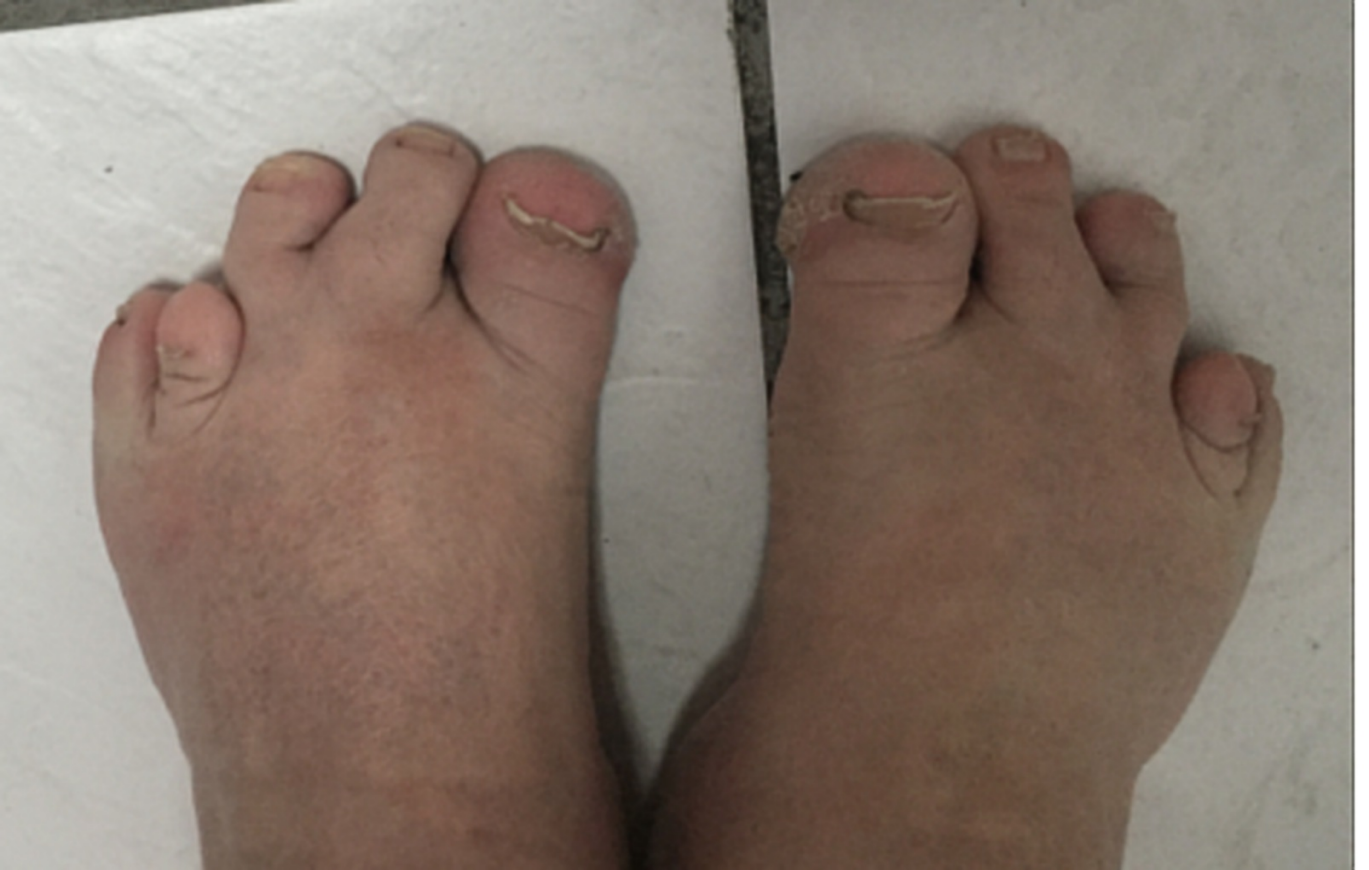
Brachydactyly of the feet Shortened first and fourth digits in both feet were observed on physical examination.

The patient in this case presented with the classic and non-classic features of Albright's hereditary osteodystrophy. His laboratories have varied with treatment; however, parathyroid hormone levels and calcium levels have been consistently elevated and depressed, respectively. The patient presented with the classic findings of pseudohypoparathyroidism such as brachydactyly of the first and fifth digits of his both hands (Figure 1), first and fourth digits of his both feet (Figure 2), together with mild intellectual disability, round facies, subcutaneous ossifications, and short stature [1]. The patient also has an anatomical short right leg. His mother also presented with the same classic findings, but with worsening gum disease. In addition, the patient presented with an exacerbation of his hypothyroidism that was diagnosed later in life as he had no documented thyroid conditions at birth. Previous TSH testing within the six month period presented with normal TSH levels; however, on this occasion the TSH was elevated to 11.390 mIU/L. Although the patient denied heat/cold intolerance, lethargy, or presence of thyroid nodules or goiter, the excessive weight gain as well as the elevated TSH levels confirmed the presence of hypothyroidism.

 

**Figure 3:**
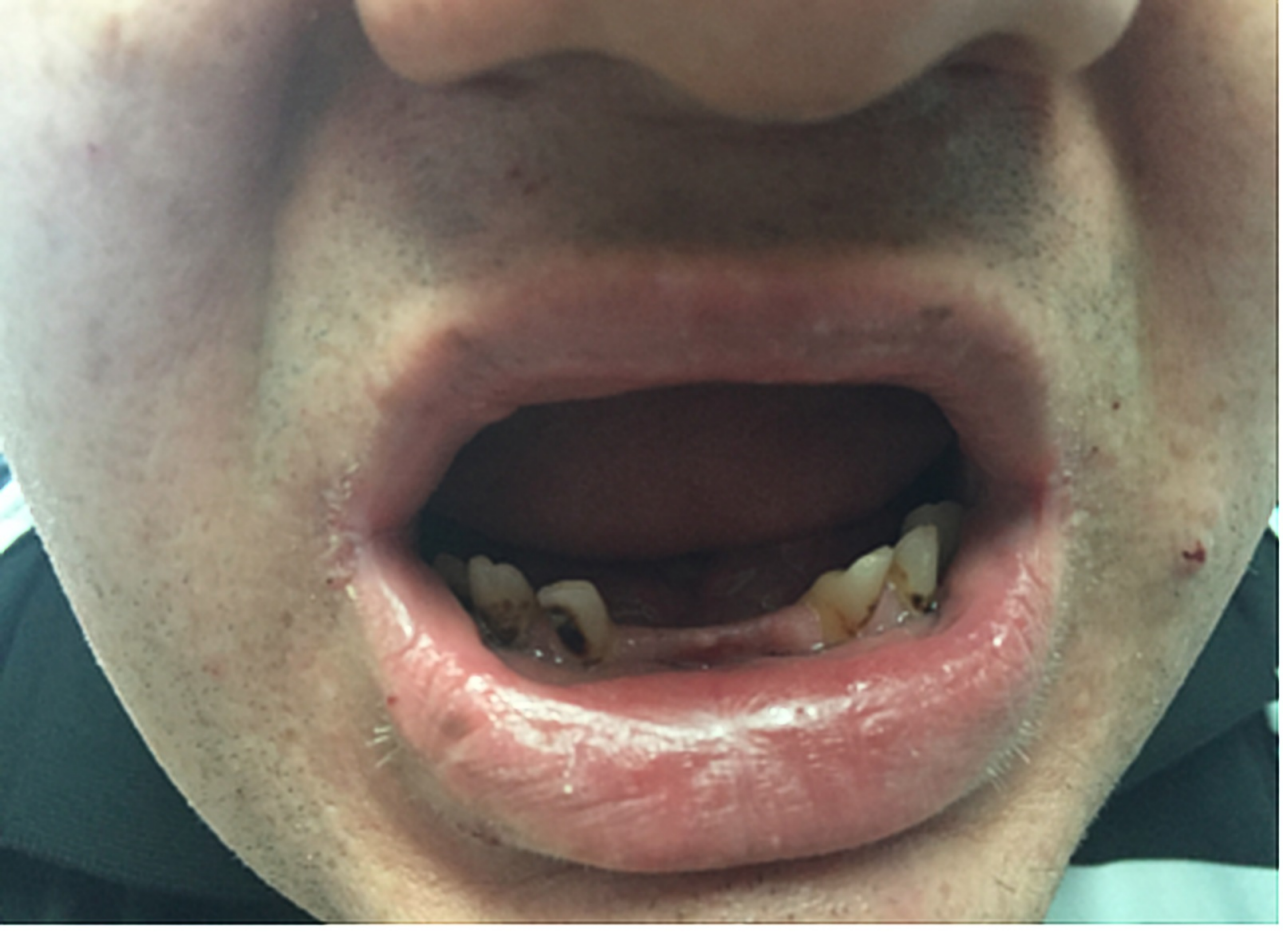
Gum disease The patient complained of severe gum disease for which he has undergone several dental extractions. As observed in this figure, the patient has several missing incisors and premolars.

Through brief review of the literature, we found that one of the studies has proposed resistance to epinephrine (a lipolysis-stimulating hormone, acting through α subunit in the stimulatory G-protein coupled receptor) as one of the mechanisms behind weight gain [4]. The study also proposed melanocortin-4 receptor (which plays a role in weight regulation) underactivity as one of the reasons behind weight gain [5]. However, both mechanisms of epinephrine resistance and melanocortin-4 receptor underactivity as the reason for weight gain in pseudohypoparathyroidism are not well established in the literature. In addition to these above-mentioned findings, the patient also presented with non-classic features of Albright's hereditary osteodystrophy. The patient had a history of carpal tunnel syndrome and bilateral sensorineural hearing loss documented in previous visits. The patient also complained of gum disease and had several missing incisors (Figure 3). While these symptoms and associated pathologies are considered non-classic, much research has concluded that these presentations are quite commonly associated with pseudohypoparathyroidism. A study performed by Joseph, et al. determined that the incidence of carpal tunnel syndrome in patients with Albright’s hereditary osteodystrophy is more common and out of the 33 patients enrolled in their study, 67% had evidence of carpal tunnel syndrome [6]. However, the study was unable to determine a cause or correlation to this relationship. In another small, retrospective investigation, researchers determined that of their subjects with pseudohypoparathyroidism, 63.6% of them had symmetric sensorineural hearing loss. The research team found that a decrease of Gs protein via Western Blot was significant in those subjects who experienced the bilateral sensorineural loss of hearing, which may explain the correlation with GNAS deficits seen in pseudohypoparathyroidism [7]. Issues associated with improper implantation of teeth in early disease and other gum related problems have been described in some case reports, but not well documented in a study of multiple patients [8]. While there has been investigation into deformities of the teeth associated with pseudohypoparathyroidism, there are relatively few cases that discuss the effect of the condition on the gums and their hygiene. Furthermore, our patient did not present with metabolic or pulmonary complications, but some research suggests that these conditions are common and should be monitored in patients with pseudohypoparathyroidism [9]. 

## Conclusions

The diagnosis and management of patients diagnosed with pseudohypoparathyroidism is complex. There are many associated symptoms and atypical presentations of the condition that clinicians could overlook. While the laboratory findings are indicative of the condition, patients with the hereditary form may present with the classic findings of brachydactyly, varying degrees of intellectual disability, obesity, round facies, subcutaneous ossifications, and short stature. However, physicians must also be wary of non-classical presentations or associated pathologies such as hypothyroidism, ear-nose-throat pathologies, and carpal tunnel syndrome. We would recommend that when a diagnosis of hereditary pseudohypoparathyroidism is made, the clinicians should use routine screenings for these non-classic conditions and associated pathologies to reduce patient morbidity.
